# Restriction of Retrotransposon Mobilization in *Schizosaccharomyces pombe* by Transcriptional Silencing and Higher-Order Chromatin Organization

**DOI:** 10.1534/genetics.116.189118

**Published:** 2016-06-22

**Authors:** Heather E. Murton, Patrick J. R. Grady, Tsun Ho Chan, Hugh P. Cam, Simon K. Whitehall

**Affiliations:** * Institute for Cell and Molecular Biosciences, Newcastle University, Newcastle upon Tyne, NE2 4HH, United Kingdom; † Biology Department, Boston College, Chestnut Hill, Massachusetts 02467

**Keywords:** *Schizosaccharomyces pombe*, *Tf2* LTR retrotransposons, higher-order chromatin organization, retrotransposition, transcriptional silencing

## Abstract

Uncontrolled propagation of retrotransposons is potentially detrimental to host genome integrity. Therefore, cells have evolved surveillance mechanisms to restrict the mobility of these elements. In *Schizosaccharomyces pombe* the *Tf2* LTR retrotransposons are transcriptionally silenced and are also clustered in the nucleus into structures termed *Tf* bodies. Here we describe the impact of silencing and clustering on the mobility of an endogenous *Tf2* element. Deletion of genes such as *set1^+^* (histone H3 lysine 4 methyltransferase) or *abp1^+^* (CENP-B homolog) that both alleviate silencing and clustering, result in a corresponding increase in mobilization. Furthermore, expression of constitutively active Sre1, a transcriptional activator of *Tf2* elements, also alleviates clustering and induces mobilization. In contrast, clustering is not disrupted by loss of the HIRA histone chaperone, despite high levels of expression, and in this background, mobilization frequency is only marginally increased. Thus, mutations that compromise transcriptional silencing but not *Tf* bodies are insufficient to drive mobilization. Furthermore, analyses of mutant alleles that separate the transcriptional repression and clustering functions of Set1 are consistent with control of *Tf2* propagation via a combination of silencing and spatial organization. Our results indicate that host surveillance mechanisms operate at multiple levels to restrict *Tf2* retrotransposon mobilization.

LTR retrotransposons are virtually ubiquitous in eukaryotes and have had major impacts upon host genome evolution, organization, and function (Kazazian 2004). They are structurally related to exogenous and endogenous retroviruses and are composed of LTR sequences that flank genes encoding, Gag, protease (PR), reverse transcriptase (RT) and integrase (IN) proteins (Beauregard *et al.* 2008). Retrotransposon RNA is synthesized by host RNA polymerase II from a promoter in the 5′ LTR. The resulting messenger RNA (mRNA) serves as a template for the translation of retrotransposon proteins and also for reverse transcription. Reverse transcription occurs within a virus-like particle and the resulting complementary DNA (cDNA) is inserted into the genome by the element-encoded IN or by homologous recombination (Beauregard *et al.* 2008). The insertion of retrotransposon cDNA is inherently mutagenic with potentially deleterious effects on the host (Levin and Moran 2011; Burns and Boeke 2012). Furthermore, the repetitive nature of retrotransposons renders them substrates for recombination and potential drivers of genome rearrangements. As a result, these elements have traditionally been viewed as harmful genomic parasites (Orgel and Crick 1980). However, there are numerous examples where host cells have domesticated transposon proteins or sequences for their own use, a process termed exaptation (Shapiro 2005; Feschotte 2008). As such, retrotransposons provide a reservoir of genetic variability (Hancks and Kazazian 2012; Chalopin *et al.* 2015).

Epigenetic controls that suppress the transcription of retroelements, play a key role in preventing their uncontrolled spread (Maksakova *et al.* 2008). DNA methylation, RNA interference (RNAi), histone modification, and chromatin remodelling have all been implicated in the suppression of specific families of LTR retrotransposons and endogenous retroviruses (Slotkin and Martienssen 2007; Maksakova *et al.* 2008; Levin and Moran 2011). However, in many cases, the controls that regulate expression of these elements are incompletely understood. Moreover, genetic studies indicate that the mobilization of LTR retrotransposons is subjected to multilayered regulation (Maxwell and Curcio 2007).

Analyses of yeast species such as *Saccharomyces cerevisiae* and *Schizosaccharomyces pombe* have provided fundamental insights into LTR retrotransposon biology (Kelly and Levin 2005; Lesage and Todeschini 2005). The genome of the common laboratory strain of *S. pombe* (972) contains a highly homogenous group of 13 *Tf2* LTR retrotransposons, which belong to the Ty3/Gypsy family (Bowen *et al.* 2003; Esnault and Levin 2015). A closely related element called *Tf1* is present in other wild strains but full-length copies of this element are absent in the laboratory strain 972 (Bowen *et al.* 2003; Esnault and Levin 2015). However, there is an extensive population of ∼250 solo LTRs in this strain, which includes sequences derived from other *Tf* families, including *Tf1*. Sequence analysis indicates that the majority of the *Tf2* elements have the potential to be active and *Tf2-12* has been shown to mobilize with a frequency of approximately two new insertions per 10^8^ cells (Sehgal *et al.* 2007). This low rate of mobilization is consistent with low levels of *Tf2* transcription in wild-type (WT) cells grown under standard conditions. Indeed, a number of studies indicate that the expression of *Tf2* retrotransposons is subjected to chromatin-mediated silencing by a variety of factors including CENP-B proteins (Cam *et al.* 2008), the Set1 histone methyltransferase (Lorenz *et al.* 2012), multiple histone deacetylases (Hansen *et al.* 2005; Durand-Dubief *et al.* 2007; Nicolas *et al.* 2007; Cam *et al.* 2008), and the histone chaperones HIRA and Asf1 (Greenall *et al.* 2006; Anderson *et al.* 2009; Yamane *et al.* 2011). Interestingly, the RNAi machinery plays only an accessory role to the exosome in this process (Cam *et al.* 2005; Hansen *et al.* 2005; Yamanaka *et al.* 2013). The role of the CENP-B homologs represents an interesting instance of exaptation, as these proteins are derived from a transposase derived from an ancient DNA transposon (Irelan *et al.* 2001). In addition to their roles in *Tf2* silencing, CENP-B and Set1 also function to cluster *Tf2* elements and solo LTRs into subnuclear structures called *Tf* bodies (Cam *et al.* 2008; Lorenz *et al.* 2012; Mikheyeva *et al.* 2014). These bodies are not apparently necessary for silencing of *Tf2* elements, but they have been proposed to prevent integration via recombination of other *Tf* elements (Cam *et al.* 2008; Mikheyeva *et al.* 2014).

In order to further investigate the host cell controls that restrict *Tf2* LTR retrotransposons, we have constructed a sensitive reporter assay that enables us to monitor the mobilization of an endogenous *Tf2* element. Rather than relying on the plasmid-encoded elements expressed from heterologous promoters that could circumvent the transcriptional controls to which endogenous native elements are subjected, this assay enabled us to determine the impact of mutations in key regulatory genes upon the mobilization frequency of an endogenous *Tf2* element. We find that mutations that compromise both transcriptional repression and also *Tf* body formation result in elevated mobilization rates. However, loss of silencing in the presence of intact *Tf* bodies is not sufficient to render high levels of mobilization. Our results, therefore, highlight that the mobility of LTR retrotransposons are subjected to regulation at multiple levels and suggest that higher-order chromatin organization is an important aspect of host cell control.

## Materials and Methods

### Strains

The *natAI* cassette was constructed by inserting a double-stranded oligonucleotide corresponding to the 37-bp intron of the *nda3*^+^ gene into the *Nru*I site of pFA6-natMX6 (Wach 1996) to give pFA6-natAI. Tagging of the *Tf2-12* element with *natAI* was achieved by assembling the following DNA fragments in pGEM-T: 0.43 kb of *Tf2-1*2 (3576–4006 bp), 1.2 kb *natAI* cassette and a 0.39-kb sequence containing the 3′ UTR and LTR of *Tf2-12* (4010–4400 bp) and 0.47 kb of chromosomal sequence downstream of *Tf2-12*. The resulting DNA fragment was released from the pGEM-T vector and used to transform strain AS50 (Sehgal *et al.* 2007). Colonies resistant to 5-FOA were isolated and correct integration of the fragment at the *Tf2-12* locus was confirmed by PCR. Two independent *Tf2-12natAI* strains were retained and used as the parental strains for mobilization assays. Mutations were introduced into the *Tf2-12natAI* background by standard genetic crosses. Strains used in this study are described in Supplemental Material, Table S1.

### *Tf2-12natAI* mobilization assays

Strains were plated onto YE5S (yeast extract 5 g/L, glucose 30 g/L, histidine, adenine, uracil, leucine and lysine hydrochloride 225 mg/L) agar to give well-dispersed single colonies. A small (<1 mm) colony was used to inoculate a 12-ml YE5S culture, which was then incubated at 30° with shaking until the culture had reached saturation (∼48 hr). A 10-ml aliquot was harvested, resuspended in 500 μl H_2_O, and plated onto two YE5S agar plates supplemented with nourseothricin (Nat) (75 μg/ml). An aliquot of the remaining culture was then subjected to 10-fold serial dilution and aliquots of the 10^−5^, 10^−6^, and 10^−7^ dilutions were plated onto YE5S agar plates. Plates were incubated at 30° for 3–4 days to allow colonies to form. The proportion of Nat-resistant cells as a fraction of the total viable cells was used to calculate *Tf2-12natAI* mobilization frequency. For each strain under analysis, the mobilization frequency of five independent cultures was measured and the median value determined. This process was repeated a minimum of three times for each strain under analysis and a mean mobilization frequency was calculated from the median values. For the WT background, the mean mobilization frequency is derived from 13 median values. *P*-values were generated by pairwise comparisons using a *t*-test.

### β-Galactosidase assays

Strains harboring a *Tf2-lacZ* reporter (Anderson *et al.* 2009) were grown in rich (YE5S) medium at 30° until they reached an OD_595_ of between 0.3 and 0.5. Cells were then harvested and processed for β-galactosidase assays as described previously (Guarente 1983). For each strain under study, the mean β-galactosidase activity was determined from at least three independent biological repeats each one assayed in duplicate. Values were scaled relative to the WT control.

### FISH analysis

FISH assays were performed as previously described (Mikheyeva *et al.* 2014). Briefly, 10 ml of cells (OD_595_ ∼0.5–1; YEA media [yeast extract 5 g/L, glucose 30 g/L, adenine 75 mg/L, uracil 225 mg/L, leucine 225 mg/L, histidine 225 mg/L and lysine hydrochloride 225 mg/L]) diluted with 10 ml of 2.4 M sorbitol YEA were cross-linked with 2.9 ml of freshly made 30% paraformaldehyde/YEA solution for 30 min in a 18° water bath shaker and subsequently quenched with 1.2 ml of 2.5 M glycine. Cells were subjected to cell wall digestion (0.5 mg/ml zymolyase solution for 1 hr) followed by RNase A treatment (0.1 mg/ml at 37° for 3 hr). Cells were hybridized with 100–150 ng dCTP-Cy3-labeled Tf2-ORF probes in 100 µl hybridization buffer (50% formamide, 2× SSC, 5× Denhart’s solution, 10% dextran sulfate) at 40° for 12–14 hr followed by washing three times with 100 µl 2× SSC for 30 min at room temperature. Nuclei were visualized by DAPI staining in 1× PBS for 5 min at room temperature. Images were obtained using a Zeiss Axioplan 2 microscope. The χ^2^-test of homogeneity was used to determine whether declustering of *Tf2* elements seen in mutant cells relative to WT was significant.

### RNA analysis

RNA was extracted using hot phenol and purified over RNase easy columns (QIAGEN, Valencia, CA) as previously described (Lyne *et al.* 2003). For strand-specific RT-PCR, one primer complementary to the sense or antisense transcript was added during first strand cDNA synthesis, while the second primer was added prior to the PCR amplification steps. cDNA for quantitative PCR (qPCR) was made using a Superscript II kit (Invitrogen, Carlsbad, CA). qPCR reactions were performed using a LightCycler 2.0 PCR system (Roche Diagnostics, Indianapolis, IN) and SYBR Green mix (Molecular Probes, Eugene, OR) using the appropriate primers.

### Southern blotting

Southern blotting of *Nco*I- and *Hind*III-digested genomic DNA was performed as previously described (Prudden *et al.* 2003). A DNA probe specific for *natAI* was amplified by PCR using pFA6a-natAI as a template and oligonucleotide primers 5ʹ-CAGAGAACAAGTACTCTAC-3ʹ and 5ʹ-TCGCCTCGACATCATCTGC-3ʹ.

### Western blotting

Western blotting was carried out as previously described (Mikheyeva *et al.* 2014). Briefly, 50 µg of protein extract was used for SDS/PAGE analysis followed by Western blotting. *Tf2* IN was detected using a rabbit antisera against *Tf1* IN at a dilution of 1:1000.

### Data and reagent availability

All strains are available upon request.

## Results

**Figure 1:**
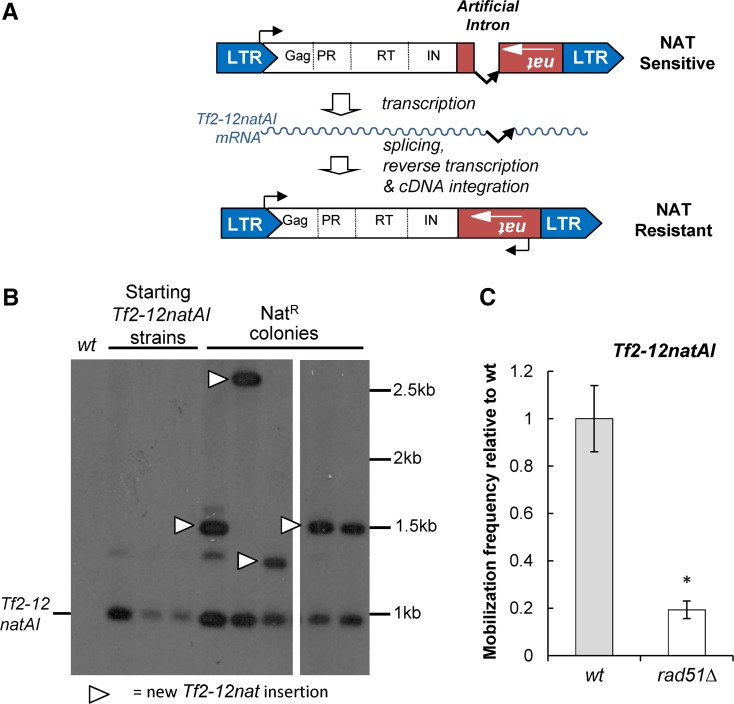
*Tf2* mobilization assay. (A) Schematic of the *Tf2-12natAI* assay. The endogenous *Tf2-12* element was marked with a *nat* antibiotic resistance cassette interrupted with an artificial intron (*AI*). Mobilization of this element results in the generation of a functional cassette and the acquisition of resistance to Nat. (B) Nat resistance (Nat^R^) arises as a result of *Tf2-12natAI* mobilization. Genomic DNA, isolated from a WT (untagged) strain, starting *Tf2-natAI* strains, and from Nat^R^ colonies was analyzed by Southern blotting with a probe specific to the *natAI* cassette. (C) Mobilization is impaired by inactivation of homologous recombination. The frequency of *Tf2-12natAI* mobilization was determined for the WT and *rad51*∆ strains by fluctuation analysis using the method of the median. Values were scaled relative to the WT. Error bars represent ± SEM. * *P* < 0.05 (*t*-test).

In order to analyze the host cell mechanisms that control the propagation of endogenous *Tf2* LTR retrotransposons, we tagged the *Tf2-12* element with an antibiotic *nat*-resistance cassette, which is disrupted with an artificial intron (*natAI*). The intron is orientated in the same transcriptional direction as that of *Tf2-12* but opposite to that of the *nat* cassette, therefore a functional *nat* cassette is generated only after successful intron splicing from the *Tf2-12natAI* transcript and integration of the processed *Tf2* element (Figure 1A). Cells that undergo a mobilization event become resistant to Nat allowing mobilization frequency to be determined from the proportion of Nat-resistant colonies in the population. Southern blotting confirmed that the acquisition of Nat resistance was accompanied by a genuine mobilization event (Figure 1B). We found that this element mobilizes with low frequency (2.06 × 10^−8^/cell) in WT cells grown under normal conditions, consistent with a previous study for an endogenous *Tf2* (Sehgal *et al.* 2007). Mobilization was also severely reduced in cells lacking the RecA homolog, Rad51(Rhp51) (Figure 1C), confirming *Tf2*’s preferred mode of genome insertion via homologous recombination rather than by IN-mediated integration (Hoff *et al.* 1998; Sehgal *et al.* 2007).

### Constitutive transcriptional activation drives *Tf2* mobilization

**Figure 2:**
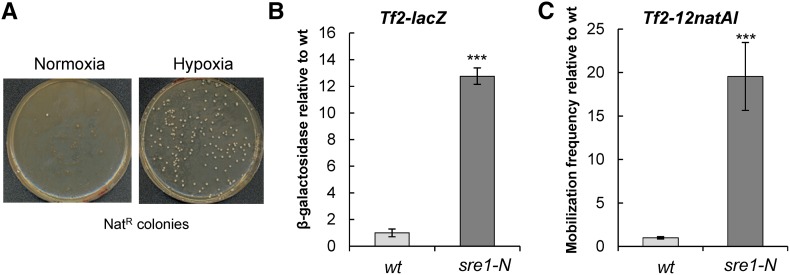
Constitutive activation of the SREBP homolog Sre1 results in high levels of *Tf2* mobilization. (A) Cells were patched onto YE5S plates and incubated at 30° for 2 days either under normal oxygen conditions or in an anaerobic jar. Cells were then resuspended in H_2_O and ∼1 × 10^8^ cells spread onto YE5S plates supplemented with Nat. Plates were incubated at 30° under normal oxygen conditions until colonies appeared. (B) The indicated strains were grown to midlog growth phase at 30° in YE5S. Cells were harvested and processed for β-galactosidase assays. Results are the mean of at least three independent assays and are scaled relative to the WT value. Error bars indicate ± SEM. (C) The mobilization frequency of *Tf2-12natAI* was determined by fluctuation analysis as described in *Materials and Methods*. Values were scaled relative to the WT. Error bars indicate ± SEM. *** *P* < 0.001 (*t*-test).

LTR retrotransposons and endogenous retroviruses are commonly quiescent under normal growth conditions but are differentially activated in response to environmental stress stimuli (Lesage and Todeschini 2005; Cho *et al.* 2008; Grandbastien 2014). Indeed, previous studies have revealed that *Tf2* elements are activated by a low oxygen environment (Sehgal *et al.* 2007) and we showed that *Tf2-12natAI* was activated under hypoxic conditions (Figure 2A). The response of *Tf2* elements to oxygen is dependent upon the transcription factor Sre1, which is an ortholog of mammalian sterol element binding protein (SREBP). Under normal oxygen conditions, Sre1 is bound to membrane in the ER but low oxygen levels result in the proteolytic cleavage of the N-terminal domain, which translocates to the nucleus and activates transcription via *SRE* elements in *Tf2* LTRs and other oxygen-responsive promoters (Sehgal *et al.* 2007; Hughes and Espenshade 2008). In order to determine whether Sre1-mediated activation is sufficient to induce mobilization, or whether additional facets of hypoxic conditions are required, we employed a strain (*sre1-N*) that expresses a constitutively active form of Sre1 (Hughes and Espenshade 2008). As expected, the *sre1-N* allele induced the expression of an integrated *Tf2-lacZ* reporter (13-fold) (Figure 2B). Furthermore, this was closely correlated with a 20-fold increase in *Tf2* mobilization (Figure 2C). Thus, the controls that restrict retrotransposon mobilization during normal growth conditions can be circumvented by active Sre1.

### Set1 methyltransferase and CENP-B homolog Abp1 restrict *Tf2* mobilization

**Figure 3:**
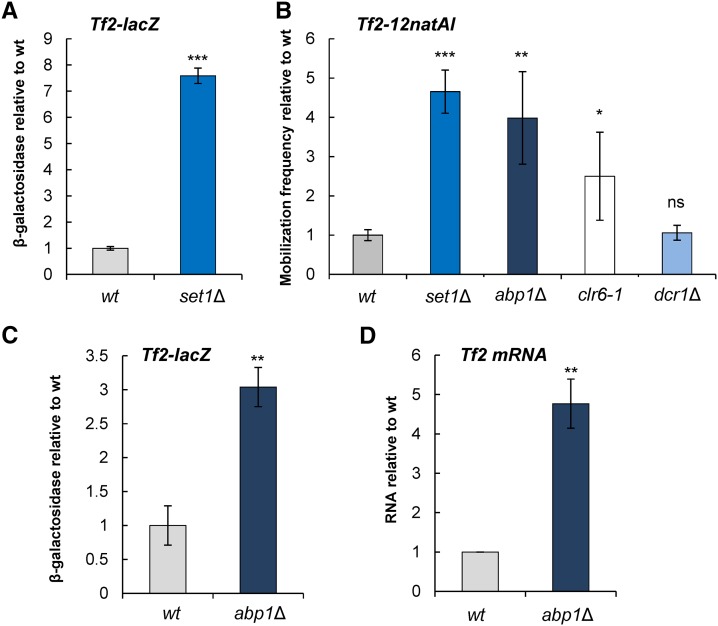
Loss of Set1- and Abp1-mediated silencing induces *Tf2* mobilization. (A) The indicated strains were grown to midlog growth phase at 30° in YES. Cells were harvested and processed for β-galactosidase assays. Results are the mean of at least three independent assays and are scaled relative to the WT value. Error bars indicate ± SEM. (B) The mobilization frequency of *Tf2-12natAI* in the indicated strain backgrounds was determined by fluctuation analysis as described in *Materials and Methods*. Values were scaled relative to the WT. Error bars indicate ± SEM. (C) As for A. (D) *Tf2* mRNA levels in the indicated strains was determined by RT-qPCR and normalized to *act1*^+^ mRNA. Values are the mean of at least three biological repeats and error bars indicate ± SEM. *** *P* < 0.001, ** *P* < 0.01, and * *P* < 0.05 (*t*-test).

Eukaryotic cells often immobilize retroelements in repressive chromatin structures, and this is believed to be a key mechanism that supresses their expression and thereby restricts their spread (Slotkin and Martienssen 2007; Maksakova *et al.* 2008). Although the expression of *Tf2* elements is repressed under normal growth conditions, these elements are found exclusively within euchromatin, which likely reflects the preference of *Tf* elements to integrate near Pol II promoters. We have previously shown that *Tf2*s are enriched with histone H3 lysine 4 methylation (H3K4me) (Noma and Grewal 2002; Cam *et al.* 2005). This euchromatin mark is mediated by Set1 and cells deficient in *set1^+^* fail to repress *Tf2*s (Lorenz *et al.* 2012). We therefore assessed the role of Set1 in the control of *Tf2* mobilization. Consistent with previous reports, deletion of *set1^+^* resulted in a significant (eightfold) increase in *Tf2-lacZ* expression and also a significant (fivefold) increase in the frequency of *Tf2-natAI* mobilization (Figure 3, A and B). We conclude that Set1 functions to both restrict the expression and the mobilization of *Tf2* elements.

Transcriptional silencing of *Tf2* elements is also dependent upon CENP-B homologs (Abp1, Cbh1, and Cbh2), which localize to *Tf2* LTRs and mediate the recruitment of class I and II HDACs (Cam *et al.* 2008; Zaratiegui *et al.* 2011; Lorenz *et al.* 2012). CENP-Bs have been shown to restrict the genomic reintegration of an “extinct” retrotransposon, *Tf1* (Cam *et al.* 2008); therefore, we investigated their roles in regulating the spread of an endogenous *Tf2* element. We determined the impact of deleting *abp1^+^*, the most prominent CENP-B member, and as previously reported, found increased expression of *Tf2* (Figure 3, C and D). Moreover the increased level of expression in the *abp1*Δ background was accompanied by a similar (∼4-fold) increase in the frequency of *Tf2-12natAI* mobilization (Figure 3B). As CENP-Bs recruit HDACs to *Tf2* LTRs, we next determined the frequency of *Tf2-12natAI* mobilization in a *clr6-1* background, which harbors a point mutation in an essential class I HDAC (Grewal *et al.* 1998). Previous studies have shown that the *clr6-1* allele is associated with a moderate derepression of *Tf2* elements (Hansen *et al.* 2005) and consistent with this finding, the frequency of *Tf2-12natAI* was modestly increased (2.5-fold) in this background (Figure 3B). Under standard growth conditions, the RNAi machinery plays only a minor role in restricting *Tf2* expression (Cam *et al.* 2005; Hansen *et al.* 2005; Yamanaka *et al.* 2013). Consistent with this, loss of the RNA processing enzyme Dcr1 did not result in increased *Tf2* mobilization (Figure 3B). Taken together, these results indicate that the propagation of *Tf2* LTR retrotransposons is restricted by the combined functions of Set1, CENP-Bs, and HDACs.

### Loss of HIRA-mediated transcriptional silencing does not result in increased *Tf2* mobilization

**Figure 4:**
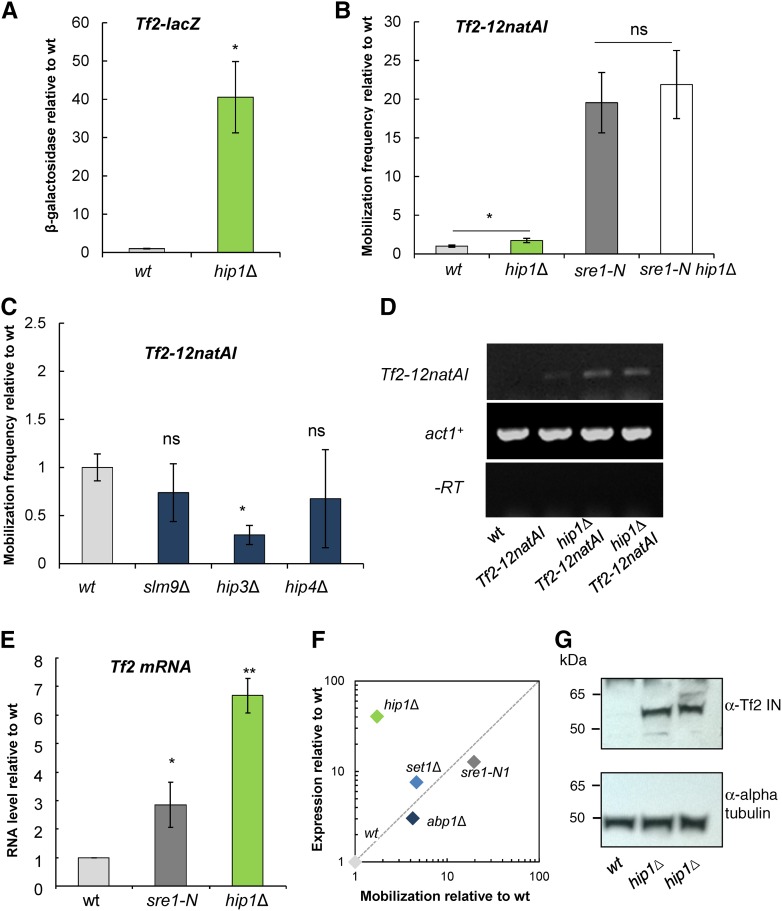
Loss of HIRA-mediated silencing does not result in uncontrolled *Tf2* element mobilization. (A) Midlog phase cells of the indicated strains were subjected to quantitative β-galactosidase assays. Mean values were determined from at least three independent assays and are scaled relative to WT. Error bars indicate ± SEM. (B) Deletion of *hip1^+^* results in only modest increase in *Tf2* mobilization. The frequency of *Tf2-12natAI* mobilization was determined for the indicated strains by fluctuation analysis using the method of the median. Values were scaled relative to the WT. Error bars represent ± SEM. Data for *sre1-N* from Figure 2C are included for comparison. (C) Deletion of other HIRA complex genes does not stimulate *Tf2* mobilization. Mobilization frequency was determined as described for B. (D) HIRA suppresses expression of the marked *Tf2-12natAI* element. RNA was prepared from the indicated strains and *Tf2-12natAI* RNA was determined by strand-specific RT-PCR. (E) Comparison of *Tf2* mRNA levels in *sre1-N* and *hip1*∆ backgrounds. RNA was prepared from the indicated strains and *Tf2* mRNA levels were assayed by RT-qPCR and normalized to *act1*^+^ mRNA. Values are the mean of at least three biological repeats and error bars indicate ± SEM. ** *P* < 0.01, * *P* < 0.05, and ns (not significant) *P >* 0.05 (*t*-test). (F) Comparison of *Tf2-lacZ* expression with *Tf2-12natAI* mobilization frequency relative to WT levels in the indicated genetic backgrounds. (G) Increased levels of *Tf2* IN in *hip1*∆ cells. *Tf2* IN in WT and two *hip1*∆ strains were detected by immunoblotting. α-Tubulin (loading control) was detected with anti-tubulin Ab (tat-1).

It has been demonstrated that the expression of all 13 *Tf2* elements is repressed by the HIRA histone chaperone complex and that loss of any one of the four HIRA complex subunits (Hip1, Slm9, Hip3, or Hip4) results in a dramatic increase in *Tf2* RNA (Greenall *et al.* 2006; Anderson *et al.* 2009; Anderson *et al.* 2010; Yamane *et al.* 2011). We therefore compared *Tf2* expression and mobilization in a background that lacks HIRA function (*hip1*Δ). Surprisingly, despite a very large increase in *Tf2-lacZ* expression (41-fold) we observed only a very modest (1.7-fold) increase in the frequency of *Tf2* mobilization in *hip1*Δ cells (Figure 4, A and B). Furthermore, deletion of the genes encoding the other HIRA complex subunits (*slm9*^+^, *hip3^+^*, and *hip4*^+^) did not result in increased *Tf2-12natAI* mobilization relative to WT cells. Indeed, mobilization was decreased in *hip3*Δ cells (Figure 4C). In order to rule out the possibility that the expression of *Tf2-12natAI* is not properly regulated by HIRA, we used strand-specific RT-PCR to measure *Tf2-12natAI* transcript levels and found that they were markedly increased in the absence of *hip1*Δ (Figure 4D). In addition, RT-qPCR revealed that in *hip1*Δ cells, *Tf2* transcripts accumulate to levels that are greater than double that of the *sre1-N* strain in which *Tf2* mobilization is dramatically increased (Figure 4E). Thus, unlike the *sre1-N*, *abp1*Δ, and *set1*Δ backgrounds, mobilization frequency does not correlate with expression in HIRA mutants (Figure 4F). One explanation of these results would be that HIRA is required for later stages of the *Tf2* life cycle such as protein maturation. Similar to *Tf1*, *Tf2* mRNA is translated as a single primary product and requires proteolytic processing by *Tf2* protease to generate mature proteins, including the IN (Levin *et al.* 1993; Hoff *et al.* 1998). To rule out that protein translation or post-translational processing of *Tf2* proteins is not somehow impaired in *hip1*Δ cells, we monitored the levels of *Tf2* IN by immunoblotting. Whereas *Tf2* integrase is almost undetectable in WT cells, its level is dramatically increased in the *hip1*Δ mutant (Figure 4G). Therefore, the absence of elevated transposition in *hip1*Δ is unlikely due to defective post-transcriptional or post-translational processing of *Tf2* products. Furthermore, deletion of *hip1^+^* did not reduce the elevated mobilization frequency observed in the *sre1-N* background (Figure 4B), which further supports the notion that loss of HIRA does not impair later stages in the *Tf2* life cycle.

### HIRA is not required for the clustering of *Tf2* elements

**Figure 5:**
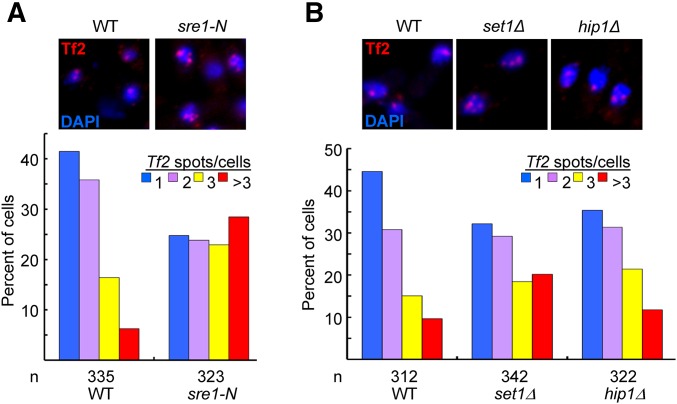
Loss of HIRA does not disrupt *Tf* bodies. (A) FISH analysis was performed using a FISH probe corresponding to the ∼3.6-kb coding region of *Tf2* elements. Representative FISH images from the indicated strains (top). Quantitative FISH analysis of observed *Tf2* foci/cell in the indicated strains (bar graph; bottom). Number of cells analyzed per strain (*n*). (B) As for A. Declustering of *Tf2*s assessed by χ^2^-test was significant in *sre1-N* and *set1*Δ (*P* < 0.001) but not *hip1*Δ (*P* > 0.05).

Our data demonstrate that loss of transcriptional silencing is not necessarily sufficient to induce *Tf2* mobilization and suggest that the propagation of these elements is subjected to additional host cell controls. A key candidate for one of these controls is higher-order chromatin organization, as these elements are physically clustered in the nucleus into structures termed *Tf* bodies (Cam *et al.* 2008). These structures are not necessary for silencing (Tanaka *et al.* 2012; Mikheyeva *et al.* 2014) but have been proposed to restrict the reintegration of *Tf2* cDNA into the genome. Importantly, *Tf2* clustering is lost in *abp1*Δ and *set1*Δ backgrounds (Cam *et al.* 2008; Lorenz *et al.* 2012) where expression and mobilization levels are well correlated. We therefore hypothesized that high levels of *Tf2* mobilization requires both increased expression and declustering. This model predicts that *Tf* bodies will be lost in the presence of active Sre1 (*sre1-N*) but retained in cells lacking HIRA (*hip1*Δ). Therefore we used a FISH assay with a probe that spans the coding region of *Tf2* to determine the status of clustering in *hip1*Δ and *sre1-N* backgrounds. As previously reported, the majority of WT cells displayed one or two *Tf2* signals consistent with these elements being assembled into *Tf* bodies. In the *sre1-N* background the proportion of cells with three or more *Tf* signals was significantly increased (*P* < 0.001), indicating that Sre1-mediated activation of *Tf2* transcription is accompanied by disruption to clustering (Figure 5A). Importantly, when *hip1*Δ cells were compared to WT, no significant increase (*P* > 0.05) in the proportion of cells with multiple *Tf2* spots was observed (Figure 5B). Therefore, despite the very high levels of expression that are associated with loss of HIRA, the sequestration of *Tf2* elements into *Tf* bodies is retained. This suggests that clustering is important for restricting the propagation of *Tf2* elements.

### Analysis of Set1 mutants suggests that *Tf2* bodies restrict mobilization

**Figure 6:**
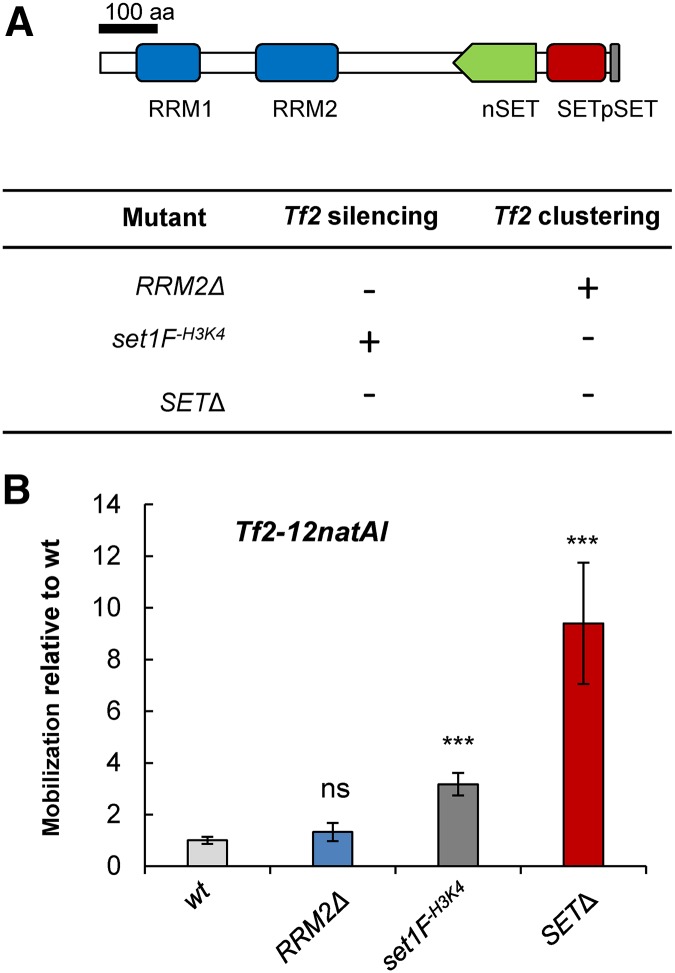
The transcriptional repression and clustering functions of Set1 suppress *Tf2* mobilization. (A, top) Schematic of the domain structure of Set1 and (bottom) a summary of the properties of the *set1* mutants (Mikheyeva *et al.* 2014). (B) Analysis of *Tf2-12natAI* mobilization frequency was determined in the indicated *set1* mutant backgrounds by fluctuation analysis using the method of the median. Values were scaled relative to the WT. Error bars represent ± SEM. *** *P* < 0.001 and ns denotes *P* > 0.05 (*t*-test).

**Figure 7:**
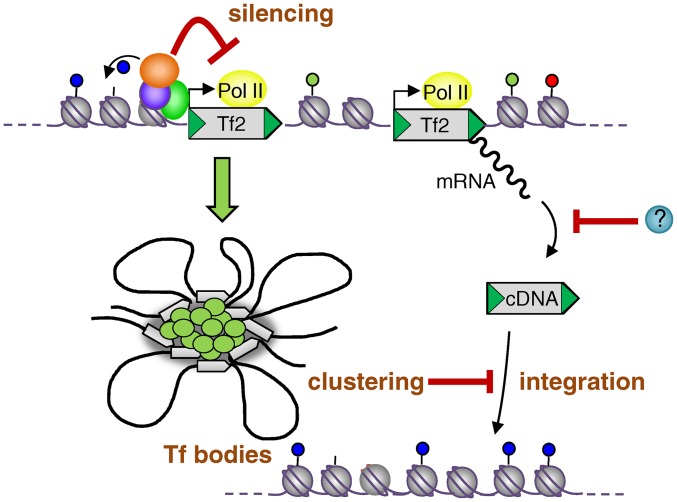
Model for the control of mobilization via transcriptional silencing and clustering into *Tf* bodies. Silencing factors such as Set1, Abp1, HIRA, and HDACs inhibit *Tf2* transcription and thereby limit cDNA accumulation. The clustering of dispersed *Tf2* elements into *Tf* bodies limits mobilization by restricting cDNA integration by homologous recombination.

In order to further dissect the roles that spatial organization and silencing play in controlling the mobilization frequency of *Tf2*, we took advantage of some separation-of-function *set1* mutant alleles. Previously it has been shown that Set1 utilizes distinct domains to repress *Tf2* expression and maintain *Tf* bodies (Mikheyeva *et al.* 2014). For example, deletion of RNA recognition motif 2 (*set1-RRM2*Δ) results in partial loss of *Tf2* repression but has only minimal disruption to *Tf* body integrity (Mikheyeva *et al.* 2014). Importantly, analysis of the *Tf2-12natAI* allele in a *set1-RRM2*Δ background revealed a low mobilization frequency that was not significantly increased relative to the WT (Figure 6, A and B). This is similar to the *hip1*Δ background, where an increase in expression in the presence of intact *Tf* bodies is apparently insufficient to elevate mobilization frequency. We next analyzed mobilization in a *set1F^-H3K4me^* background. This allele encodes a mutant protein that represses *Tf2* expression but is unable to mediate either H3K4 methylation or *Tf* body maintenance (Mikheyeva *et al.* 2014). In this background, we found that mobilization frequency was modestly increased (threefold) relative to the WT, again suggesting that *Tf* bodies restrict the propagation of these elements. As predicted, a high mobilization frequency (ninefold relative to WT) was observed in the *set1-SET*Δ mutant, which lacks both transcriptional repression and *Tf* body maintenance functions (Mikheyeva *et al.* 2014). Taken together, these results indicate that *Tf2* LTR retrotransposons are subjected to control at multiple levels and suggest that spatial organization functions to restrict their mobility. We therefore propose that the alleviation of transcriptional silencing and disruption of *Tf* bodies is necessary for high levels of element mobilization (Figure 7).

## Discussion

Here we compared the impact of mutations upon both the expression and mobilization frequency of fission yeast *Tf2* LTR retrotransposons. Our results indicate that host controls of the *Tf2* life cycle operate at multiple levels and suggest that transcriptional silencing and higher-order chromatin organization cooperate to restrict the mobility of these elements.

That an increase in *Tf2* mRNA is not necessarily accompanied by a proportional increase in mobilization indicates the existence of post-transcriptional controls. Post-transcriptional control of LTR retrotransposition has also been revealed in *Arabidopsis* as abolition of DNA methylation in *met1* mutants does not increase the mobilization of retrotransposons despite their widespread transcriptional activation (Mirouze *et al.* 2009). Genetic analysis of the *Évadé* (*EVD*) *copia*-type LTR retrotransposon demonstrated that following transcriptional reactivation, subsequent steps in its lifecycle are suppressed by the plant-specific RNA polymerases IV/V and the histone methyltransferase KRYPTONITE. However while *EVD* mobilization is stimulated by the loss of these regulators, these mutations do not affect the mobilization of other potentially active retrotransposons (Mirouze *et al.* 2009). Therefore post-transcriptional controls of retrotransposition may be individually tailored to specific elements.

Our analyses implicate subnuclear organization of *Tf2*s in the control of their mobilization. The fission yeast nucleus, similar to those of higher eukaryotes, is segregated into a variety of distinct chromosomal territories and domains and the importance of this organization genome function is becoming increasingly apparent (Nunez *et al.* 2009; Zhao *et al.* 2009; Tanizawa *et al.* 2010; Mizuguchi *et al.* 2014). *Tf2* elements are subjected to a high degree of organization as they are clustered into bodies that are localized in close proximity to centromeres at the nuclear periphery (Cam *et al.* 2008; Tanaka *et al.* 2012). In these respects, *Tf2* retrotransposons exhibit similarities with HIV-1 provirus in latently infected lymphocytes, which is also found associated with centromeric heterochromatin at the nuclear periphery (Dieudonne *et al.* 2009). In the case of HIV-1, nuclear positioning has been correlated to expression because transcriptional induction results in the loss of proviral association with heterochromatin, although localization at the nuclear periphery is retained (Dieudonne *et al.* 2009). Furthermore, it has been shown that inactive HIV-1 provirus is found in close proximity to PML bodies and that transcriptional activation requires displacement from these subnuclear structures (Lusic *et al.* 2013). However, in the case of *Tf2* elements, transcriptional silencing can be separated from their spatial organization. The *set1F^-H3K4me^* mutant allele abolishes clustering but does not increase *Tf2* expression (Mikheyeva *et al.* 2014). Also loss of Ku function (*pku70*Δ or *pku80*Δ) compromises interaction of *Tf2* elements with centromeres and the nuclear periphery but does not impair the transcriptional silencing of these elements (Tanaka *et al.* 2012). Furthermore, we show here that *Tf2* silencing can be circumvented without disruption to *Tf* bodies. We conclude therefore that the spatial organization of *Tf2* elements does not restrict their mobility via an impact upon transcriptional silencing. Instead, we propose that *Tf* bodies restrict other steps in the retrotransposon life cycle (Figure 7).

*Tf* bodies have the potential to restrict the integration of cDNA into the genome, particularly as *Tf2* elements prefer to mediate this step by homologous recombination into an existing element, a process which is termed “integration site recycling” (Hoff *et al.* 1998). Superficially, this may appear to be somewhat of a futile cycle. However, it has advantages in that it avoids integration into a region of the genome that is harmful to the host while still allowing element evolution (Hoff *et al.* 1998). Sequestration of these elements into *Tf* bodies may provide an environment that restricts their accessibility to suppress cDNA recombination. Consistent with this, *abp1*Δ *cbh1*Δ CENP-B double mutants, in which clustering is absent, have elevated levels of DNA recombination-associated Rad22 foci (homologous to *S. cerevisiae* Rad52) at LTRs (Zaratiegui *et al.* 2011). *Tf* bodies may also prevent recombination between different *Tf2* elements, thereby suppressing potentially harmful chromosome rearrangements. Interestingly, it has been proposed that the compact chromatin conformation of *S. cerevisiae Ty* elements suppresses recombination hotspot activity and thus prevents potentially harmful exchange between these repeated sequences (Ben-Aroya *et al.* 2004). Nonetheless, *Tf* bodies could suppress alternative steps in the retrotransposon life cycle, such as RNA processing and export. There is clear precedent for the ability of chromatin structure to influence RNA processing steps (Mathieu and Bouche 2014), and furthermore, links between nuclear bodies and a variety of RNA processing events have been well documented (Morimoto and Boerkoel 2013). Therefore, it is possible that *Tf* bodies could provide a molecular trap that restricts *Tf2* mRNA processing and/or export. Arguing against this, levels of *Tf2* IN were found to be markedly increased in the *hip1*Δ mutant, which retains *Tf* bodies. This suggests that *Tf* bodies do not prevent the export of *Tf2* mRNA from the nucleus.

Transcriptional silencing is a common host cell response to transposable elements and is considered to be pivotal to controlling their activity. Our findings suggest that fission yeast cells can also restrict the mobilization of retroelements at a post-transcriptional level through epigenetic control of their nuclear organization. As such, it will be important to determine whether, and to what extent, the activity of retrotransposons in other systems is controlled by higher-order organization of chromatin.
